# Axl in ovarian cancer: a step forward for clinical breakthrough?

**DOI:** 10.18632/oncotarget.13457

**Published:** 2016-11-14

**Authors:** Marialuisa Sensi, Silvana Canevari, Antonella Tomassetti

**Affiliations:** Department of Experimental Oncology and Molecular Medicine, Fondazione IRCCS Istituto Nazionale dei Tumori, Milan, Italy

**Keywords:** Axl, epithelial ovarian cancer, tumor progression, prognosis, targeted therapy

The receptor tyrosine kinase (RTK) Axl has been shown to be involved in cancer progression [1]. A systematic search in PubMed by combining Axl and cancer keywords retrieved about 500 publications being more than 400 published from 2010. The majority are basic-translational reports aiming to clarify Axl functional role in both haemathological and solid malignances. Beside its contribution to proliferation and escape from apoptosis, Axl activation resulted deeply involved in increased invasiveness, metastasis formation and chemorestance [1]. Numerous studies highlighted the possible exploitation of Axl as a prognostic marker for outcome as well as a new therapeutic target either alone or in combination with other targeting agents. Despite the interest of medicinal chemistry and pharma companies in developing Axl-targeted inhibitors, the number of registered and recruiting clinical trials is so far limited to six (https://clinicaltrials.gov/), two of which, in Phase II, with multi-kinase inhibitors (Cabozantinib and Crizotinib). Since Axl is expressed at different levels in most advanced tumors, the selection of the patients who would benefit of anti-Axl therapies remains the major challenge.

**Figure 1:**
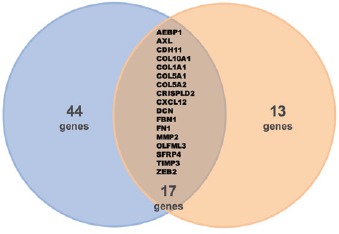
Venn diagram between the two published Axl signatures [4, 6] highlights a common 17-gene signature

We have recently reported that Axl and Axl-co-regulated genes could stratify melanoma and epithelial ovarian cancer (EOC) subtypes [2-4]. In melanoma we showed that Axl expression was associated with poorly differentiated tumors [2]. Among those, co-expression of Axl and EGFR and lack of expression of ERBB3 mark a melanoma subtype, with a peculiar molecular signature, that display intrinsic resistance to BRAF inhibitors [3]. In 2015 we provided evidence that Axl is a molecular determinant of aggressiveness for the most frequent EOC type (high grade EOC, HGEOC) [5]. The novelties of our report are: i) the identification of a novel signalling pathway elicited by ligand-dependent Axl activation; ii) the identification, by analyzing the gene expression profiles of a total of 976 HGEOC patients, of a geneset comprising genes whose expression resulted co-regulated with that of Axl; iii) the assessment that this gene signature, up-regulated in the ‘mesenchymal’ EOC subtype, is able to identify EOC patients with the shortest overall survival. More recently, the capability of Axl-co-expressed genes to characterize the most aggressive HGEOCs was confirmed by Antony et al. analyzing gene expression data of 1538 HGEOC patients [6] and 17 genes proved to be common between the two Axl-signatures (Figure 1) [3, 6]. As observed in other carcinomas [1], in mesenchymal EOC cell lines, upon activation by its ligand GAS6, Axl co-clustered with and transactivated the RTKs cMET, EGFR, and HER2, producing sustained extracellular signal-regulated kinase (ERK) activation. This described cross-talk provide further evidence that Axl participates to signalling network/s constituted by RTKs [1-3, 5] or by other relevant molecules as integrins [4], activated in the most aggressive EOCs.

Thus, data obtained from two independent laboratories converge on the notion that Axl together with 16 genes are highly expressed in the most aggressive EOCs. Although this core list of common co-regulated genes deserves validation and refinement in ‘mesenchymal’ subtype of HGEOCs, these findings support further investigation of these genes. Indeed, in a pathological context, Axl protein expression by immunohistochemistry resulted almost ubiquitous among HGEOCs [3]; thus the possibility to analyze co-expressed proteins offers a valid and promising approach for prognostic and predictive tools in a clinical setting where no stratifying biomarker currently exists.

Altogether these data also reinforce the idea that Axl appears a fascinating candidate for targeted therapies in the mesenchymal subtype of HGEOC patients. However, we should be aware that Axl is also involved in the regulation of the immune system. Therefore, Axl inactivation could exert a dual effect: one direct, as anti-tumor agent, and the other indirect, as modulator of the immune response which can lead to either positive or negative effect on tumor cells. While further analysis will be required to investigate these particular aspects, the targeting of Axl downstream signalling networks would improve selectivity providing cancer patients with new therapeutic options for this aggressive form of EOC.

As final note, a recent observation indicates that Axl expression and activation have a positive role in the homologous recombination (HR)-mediated DNA repair and its inhibition increases the efficacy of PARP inhibitor on carcinoma cells of different histotypes [7]. Since PARP inhibitors have shown efficacy in HR deficient HGEOC [5], these findings lead to the hypothesis that the combination of Axl and PARP inhibitors could elicit a synthetic lethal effect in AXL over-expressing tumors of the ‘mesenchymal’ subtype.
